# A Case of Sevoflurane Use during Pregnancy in the Management of Persistent Status Asthmaticus

**DOI:** 10.1155/2017/3547242

**Published:** 2017-05-07

**Authors:** Jessica Parrott, Mitch Tener, Katie Dennis, Matthew Sharpe, Cecily Clark-Ganheart

**Affiliations:** ^1^Division of Maternal Fetal Medicine, Department of Obstetrics and Gynecology, University of Kansas School of Medicine, 3901 Rainbow Boulevard, Kansas City, KS 66160, USA; ^2^Department of Pulmonary and Critical Care Medicine, University of Kansas School of Medicine, 3901 Rainbow Boulevard, Kansas City, KS 66160, USA; ^3^Department of Pathology and Laboratory Medicine, University of Kansas School of Medicine, 3901 Rainbow Boulevard, Kansas City, KS 66160, USA

## Abstract

**Background:**

Sevoflurane is rarely used for the treatment of status asthmaticus. We report a case of sevoflurane hepatotoxicity in pregnancy with presentation similar to HELLP syndrome.

**Case:**

A G2P1001 at 23 weeks in status asthmaticus presented with pCO2 > 130 and pH < 7. She was nonresponsive to traditional therapy. Sevoflurane was added for a 24 hr period. Respiratory status improved. Extubation occurred on day 12. Workup for preeclampsia spectrum disorders occurred due to maternal hypertension. Given the atypical presentation and hepatotoxicity, a liver biopsy was performed. Histologic features suggested drug induced hepatic injury. Liver function subsequently normalized. She delivered a term neonate without short-term complications.

**Conclusion:**

The use of sevoflurane is a treatment option of status asthmaticus during pregnancy. Providers should be aware of the potential for hepatotoxicity.

## 1. Introduction

Status asthmaticus is a severe asthma exacerbation that is refractory to bronchodilator and corticosteroid treatment. Standard treatment often employed includes nebulized bronchodilators, magnesium, corticosteroids, and mechanical ventilation. Additional considerations, although rarely used, include sedatives, neuromuscular blockade, or inhaled anesthetics [1]. Case reports of sevoflurane use in emergency departments and the pediatric population exist for the treatment of status asthmaticus prior to extracorporeal membrane oxygenation (ECMO) [2]. However, data regarding the use during pregnancy is limited. Matters are further complicated by the potential of hepatotoxicity with use of halogenated inhaled anesthetics. With informed patient consent, we present a case of refractory status asthmaticus during pregnancy with resultant hepatotoxicity.

## 2. Case

A 31-year-old, gravida 2 para 1001, patient was transferred to our medical ICU at 23 2/7 weeks of gestation with status asthmaticus complicated by acute hypercarbic respiratory failure. She was intubated and paralyzed with a pH < 7.0 and pCO2 > 130. The picture was further complicated by marked bronchoconstriction with elevated PEEP and increased airway resistance nonresponsive to nebulized beta agonists, glucocorticoid, magnesium, and epinephrine treatments. She was initially stabilized on continuous nebulizers, magnesium, propofol, ketamine, and inhaled helium-oxygen (heliox) mixture. Due to persistent difficulty with oxygenation and limited ventilation over a 24-hour period, ECMO was considered: during this time, the patient's oxygen saturation was 85–94% with arterial pH 6.8–7.2. However, due to the potential complications associated with ECMO use during pregnancy, a decision was made to add an inhaled anesthetic for its bronchodilator effects. The patient was transitioned to an anesthesia ventilator system that could provide heliox with inhaled anesthetic. Sevoflurane was initiated, allowing us to lower our peak pressures and maintain oxygen saturations >95%. During this time, the patient was noted to have elevated blood pressure (137–166/70–82 mmHg) requiring intravenous treatment. A 24-hour urine protein was found to be elevated at 739 mg. On review of the patient's previous records, it was determined that the patient was likely a chronic hypertensive with history of antihypertensive medication use and no further workup was undertaken.

Over the next 24 hours, the patient was noted to have improved oxygenation and the sevoflurane was discontinued. She was transitioned back to a traditional ventilator. She remained hypercapnic but maintained a normal pH while on heliox, steroids, propofol, and ketamine. The following day, the patient was noted to have an acute rise in her liver enzymes with an AST of 91 U/L and ALT of 32 U/L; platelets at that time were 243 K/UL. The liver enzymes were noted to progressively rise over the next 10 days while the platelet count remained normal (196–316 K/UL).

The patient's respiratory status continued to slowly improve during this time as she was weaned off of the paralytic, corticosteroids, and ketamine. We were able to decrease her peak pressures and elevated PEEP. She was ultimately extubated on hospital day 10. Due to altered mental status, she was reintubated. CT head was negative. Her laboratory evaluation at that time showed AST 161 U/L, ALT 329 U/L, Cr 0.28 mg/dL, Hgb 9.4 g/dL, Plt 261 K/UL, LDH 1029 U/L, and 24-hour urine protein 1245 mg. Diagnosis of atypical hemolysis, elevated liver enzymes, and low platelet count syndrome (HELLP syndrome) was considered a possibility. However, due to her periviable gestational age, hematology and hepatology were consulted to rule out other potential etiologies. An extensive autoimmune and infectious workup was done that included antinuclear antibody, anti-mitochondrial antibody, anti-smooth muscle antibody, ADAMTS-13, acute and chronic hepatitis panels, Epstein-Barr virus, cytomegalovirus, herpes simplex virus, and human herpes virus 6, all of which were unremarkable. Of note, the maternal Rhesus status was negative. Given that maternal blood transfusion did not occur, Rh incompatibility was unlikely to explain the maternal findings. Abdominal ultrasound was performed and noted to be within normal limits. The patient was reextubated 2 days later.

**Figure 1:**
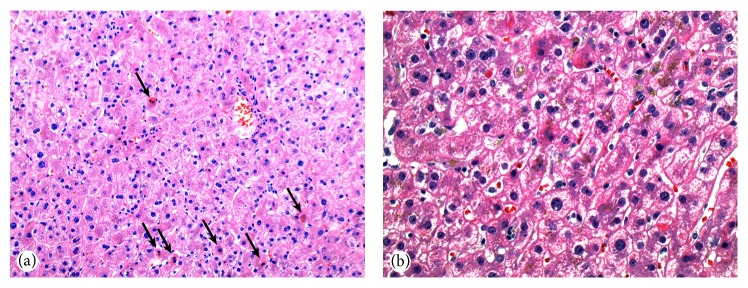
Liver biopsy. (a) Hematoxylin and eosin ×20: liver biopsy specimen showing lobular disarray with numerous apoptotic hepatocytes (arrows) and increased sinusoidal inflammatory cells. No steatosis or frank necrosis is present. (b) Hematoxylin and eosin ×40: liver biopsy specimen showing lobular disarray, prominent apoptotic hepatocytes, and biliary stasis, compatible with recent drug induced liver injury.

On hospital day 15, the patient's AST peaked at 1146 U/L and ALT peaked at 3168 U/L. Alkaline phosphatase and total bilirubin remained within normal limits. A liver biopsy showed acute hepatitis with prominent apoptotic hepatocytes, cholestasis, and increased sinusoidal inflammatory cells (Figure 1). Immunostains for herpes simplex virus I and II and in situ hybridization for Epstein-Barr virus were negative. There was no significant steatosis or fibrosis, frank necrosis, or fibrin deposition. Given the absence of features of an underlying chronic liver disease and a negative viral and autoimmune hepatitis workup, the findings were consistent with acute drug induced liver injury, most likely due to sevoflurane exposure. Her respiratory status continued to improve and she was discharged from the hospital. No additional episodes of hypertension were noted. Liver enzymes returned to normal over a two-month period (AST 16 U/L and ALT 7 U/L) and repeat 24-hour urine protein was 188 mg two months after the episode. With prolonged periods of maternal hypoxia during the initial 24 hours of hospitalization, there was concern for fetal hypoxic encephalopathy. However, a fetal MRI performed four weeks after the inciting maternal event failed to demonstrate evidence of hypoxic insult. The patient was induced at term due to new-onset oligohydramnios. A healthy term neonate was delivered via spontaneous vaginal delivery without short-term complications despite prolonged maternal hypoxia, acidemia, and permissive hypercarbia.

## 3. Discussion

Severe, acute asthma generally leads to significant air trapping, hyperinflation, decreased venous return, and potentially detrimental hemodynamic consequences. The mainstay of treatment revolves around the administration of inhaled bronchodilators and systemic glucocorticoids. The decision to intubate a patient with a severe asthma exacerbation is based upon clinical judgment in conjunction with arterial blood gas measurement. As a general rule, if the patient demonstrates progressive fatigue, continued work of breathing, or alterations in consciousness, intubation is indicated [3]. These patients often will also require deep sedation and paralysis to prevent further complications. In life-threatening exacerbations, such as the patient presented here, nontraditional therapies may be utilized. This can include a helium-oxygen mixture to decrease airflow resistance, magnesium sulfate for bronchodilatory effects, ketamine for potential bronchodilatory effects, or inhalational anesthetics [3]. The mechanism of action for inhaled anesthetics is unclear, but it is thought that these agents have direct bronchodilatory effects on the airways and an increase in cholinergic tone. Halothane, isoflurane, and sevoflurane have been shown to be effective in case reports [2]. Hypotension is usually the limiting factor with use of these agents and bronchoconstriction can be seen after discontinuation of the medications. Additionally, when maternal ventilation is suboptimal, a concern for fetal hypoxemia exists. In our case, particularly due to gestational age, our focus was improving maternal ventilation as opposed to delivery of a periviable neonate. While cordocentesis is an option to assess fetal oxygen saturation, we felt the clinical benefit would be minimal in this situation. Additionally, in the presence of maternal academia, the fetal oxygen disassociation curve would experience a left-ward shift allowing for increased amounts of oxygen delivery to the neonate despite fetal acidosis.

HELLP syndrome, a disorder marked by hemolysis, elevated liver enzymes, and low platelets, has a typical onset in the third trimester. It can be difficult to differentiate HELLP syndrome from other disease processes due to multiorgan involvement. The differential diagnoses include acute fatty liver of pregnancy, thrombotic thrombocytopenic purpura, hemolytic uremic syndrome, systemic lupus erythematosus, and antiphospholipid syndrome [4]. At this gestational age, maternal mortality is reported at ~1% with perinatal mortality rate of 7–22% [5]. Rare case reports of HELLP syndrome occurring at periviable gestational age have been reported in the literature [6–9], all associated with triploidy, molar pregnancy, or antiphospholipid syndrome. It is important to ensure that the diagnosis of HELLP syndrome is accurate during the periviable period as it has significant implications on pregnancy outcome. Continuing the pregnancy may result in significant maternal morbidity/mortality with severe end organ damage but treatment requires delivery of a fetus who will require a prolonged NICU admission and will face high rates of morbidity and mortality. As such, when a patient presents with hepatic dysfunction at an early gestational age, a thorough assessment of hepatotoxicity should include consideration of disorders beyond the spectrum of hypertensive disorders of pregnancy. Furthermore, evaluation via liver biopsy may assist with making an accurate diagnosis and guiding appropriate treatment.

In our case, a thorough evaluation by hepatology and hematology was performed, including a negative viral and autoimmune workup. Eventually a liver biopsy was performed, and the histologic features were consistent with drug induced livery injury. The typical findings of HELLP syndrome including frank necrosis, periportal hemorrhage, and fibrin deposition were not observed [5]. Sevoflurane, the halogenated inhaled anesthetic used to treat the patient's status asthmaticus, was the presumed inciting agent. Review of the literature has rare reports of sevoflurane-associated hepatotoxicity, primarily in Japanese literature [10]. The patient's overall state of persistent acidosis likely made her more susceptible to hepatotoxicity. Older halogenated anesthetics cause hepatotoxicity via production of trifluoroacetic acid (TFA) that subsequently produce trifluoroacetylated components and cause an immunogenic response in the patient, resulting in hepatitis. Compared to these older halogenated anesthetics, sevoflurane has low hepatotoxic potential as it is metabolized into hexafluoroisopropanol (HFIP) rather than TFA. HFIP has less protein binding capacity, does not accumulate, and is rapidly converted into a stable nontoxic compound that is excreted in the urine. Ultimately, its low hepatotoxic potential makes it the preferred inhaled anesthetic for patients with hepatic disease or at risk for postoperative hepatic injury; however, the risk of hepatotoxicity from TFA still exists [11].

The use of sevoflurane for the treatment of persistent status asthmaticus is an option during pregnancy. Should failure of traditional therapy occur, the addition of sevoflurane has the potential to improve the patient's clinical course. Practitioners should remain aware of the potential of hepatotoxicity, particularly in the presence of acidosis.
